# Supplementary material for: Synthesis and Bioactivity of Thiazolethioacetamides as Potential Metallo-β-Lactamase Inhibitors
Source: Antibiotics (Basel). 2020 Feb 26;9(3):99. doi: 10.3390/antibiotics9030099 (PMC7175138; doi:10.3390/antibiotics9030099)
Supplement: Supplementary file 1 [file antibiotics-09-00099-s001.zip › supporting information/Supporting Information.doc]

**Supporting Information**

Synthesis and Bioactivity of Thiazolethioacetamides as Potential Metallo-β-Lactamase Inhibitors

YiLin Zhang^1,*^, Yong Yan^1^, Xuejun Wang^1^, Kewu Yang^2^

**Table of Contents**

**Synthetic Experimental Procedures**	2

General	2

Synthesis and characterization	2

**^1^H and ^13^C NMR data of halogen-substituted triazolethioacetamides**	3

1. N-(4-chlorophenyl)-5-(5-phenyl-4H-1,2,4-thiazolyl)thioacetamide	3

2. N-(3-chlorophenyl)-5-(5-phenyl-4H-1,2,4-thiazolyl)thioacetamide	3

3. N-(3,4-chlorophenyl)-5-(5-phenyl-4H-1,2,4-thiazolyl)thioacetamide	3

4. N-(2,4-dichlorophenyl)-5-(5-phenyl-4H-1,2,4-thiazolyl)thioacetamide	3

5. N-(4-chloro-2-nitrophenyl)-5-(5-phenyl-4H-1,2,4-thiazolyl)thioacetamide	3

6. N-(5-chloro-2-nitrophenyl)-5-(5-phenyl-4H-1,2,4-thiazolyl)thioacetamide	4

7. N-(2-chloro-4-nitrophenyl)-5-(5-phenyl-4H-1,2,4-thiazolyl)thioacetamide	4

8. N-(4-(trifluoromethoxy)phenyl)-5-(5-phenyl-4H-1,2,4-thiazolyl)thioacetamide	4

9. N-(4-fluorophenyl)-5-(5-phenyl-4H-1,2,4-thriazolyl)thioacetamide	4

10. N-(3-fluorophenyl)-5-(5-phenyl-4H-1,2,4-thiazolyl)thioacetamide	4

11. N-(2-fluorophenyl)-5-(5-phenyl-4H-1,2,4-thiazolyl)thioacetamide	5

12. N-(2,6-difluorophenyl)-5-(5-phenyl-4H-1,2,4-thiazolyl)thioacetamide	5

# Synthetic Experimental Procedures

## General

General chemicals were purchased from TCI and were used without further purification. All antibiotics used were purchased from Sigma-Aldrich.^1^H NMR and ^13^C NMR spectra were recorded with a Bruker DRX 600 MHz spectrometer. The peaks patterns are indicated as follows: s, singlet; d, doublet; t, triplet; q, quartet; dd, doublet doublet; m, multiplet. The spectra were recorded with TMS as internal standard. Coupling constants (J) were reported in hertz (Hz). Chemical shifts were given in part per million (ppm) on the delta scale. Analytical Thin Layer Chromatography (TLC) was carried out on silica gel F_254_ plates with visualization by ultraviolet radiation. HRMS spectra were recorded on a Bruker MicrOTOF-Q II mass spectrometer. Inhibition studies were performed on an Agilent-8453 UV-visible spectrometer.

## Synthesis and characterization

The synthetic route of the twelve thiazolethioacetamides was shown in Scheme 1. N-substituted-2 -chloroacetamides (**N 1-12**) were first prepared by acylation between substituted anilines and chloroacetyl chloride. The methyl-2-hydroxybenzoate (**s1**) were prepared by esterifying of [salicylic](../../../../Program%20Files%20(x86)/Youdao/Dict/8.3.1.0/resultui/html/index.html#/javascript:;) [acid](../../../../Program%20Files%20(x86)/Youdao/Dict/8.3.1.0/resultui/html/index.html#/javascript:;) and converted into hydrazides **s2** by condensing with hydrazine. The hydrazides reacted with CS_2_ under basic condition to give **s3**, and then stirred with H_2_SO_4_ in [ice-water](javascript:;) [bath](javascript:;) to afford 2-(5-mercapto-1,3,4-thiadiazol-2-yl)phenol (**s4**).

**Scheme 1. Synthetic route of halogen-substituted thiazolethioacetamides**

A solution of 2-(5-mercapto-1,3,4-thiadiazol-2-yl)phenol (**s4**) (3 mmol) and K_2_CO_3_ (3.6 mmol) dissolved in H_2_O (15 mL) were added in a 50 mL three-neck round bottomed flask, kept stirring for 30 min. After N-substituted-2-chloroacetamides (**N1-12**) (3 mmol) dissolved in hot ethanol (5 mL) was added drop wise, the reaction mixture was heated to reflux for 6 h. Reaction mixture was cooled to RT and neutralized with 5 M HCl to a pH approximately 7.0. The resulting white solid was collected by filtration, washed with H_2_O repeatedly (3×80 mL) and dried in vacuo to obtain **1-12**.

**^1^H and ^13^C NMR data of halogen-substituted [triazole](../../../../Program%20Files%20(x86)/Youdao/Dict/8.3.1.0/resultui/html/index.html#/javascript:;)thioacetamides:**

**1. N-(4-chlorophenyl)-5-(5-phenyl-4H-1,2,4-thiazolyl)thioacetamide (1)**

White powder, yield 72.5 %, ^1^H NMR (600 MHz, DMSO-d_6_): δ 10.90 (s, 1H), 7.84 (d, J = 8.2 Hz, 1H), 7.61 (d, J = 8.6 Hz, 2H), 7.44 (d, J = 8.6 Hz, 2H), 7.42-7.36 (m, 2H), 6.97 (d, J = 8.3 Hz, 1H), 4.23 (s, 2H).^13^C NMR (151 MHz, DMSO-d_6_): δ 171.11, 168.63, 163.32, 157.74, 133.73, 133.41, 130.29, 129.46, 129.28, 122.69, 119.42, 117.14, 114.91, 33.30. HRMS [M+Na]^+^ (m/z) for C_17_H_13_ClN_2_O_2_S_2_: calcd. 399.9957, obsd. 399.9861.

**2. N-(3-chlorophenyl)-5-(5-phenyl-4H-1,2,4-thiazolyl)thioacetamide (2)**

White powder, yield 68.1 %, ^1^H NMR (600 MHz, DMSO-d_6_): δ 11.32 (s, 1H), 7.86 (s, 1H), 7.56 (s, 3H), 7.41 (s, 2H), 7.00 (s, 1H), 6.94 (s, 1H), 4.23 (s, 2H). ^13^C NMR (151 MHz, DMSO-d_6_): δ 171.10, 163.31, 163.03, 157.98, 136.21, 133.49, 133.20, 130.83, 129.34, 128.93, 128.50, 127.40, 119.27, 117.21, 116.49, 33.34. HRMS [M+Na]^+^ (m/z) for C_17_H_13_ClN_2_O_2_S: calcd. 399.9957, obsd. 399.9870.

**3. N-(3,4-dichlorophenyl)-5-(5-phenyl-4H-1,2,4-thiazolyl)thioacetamide (3)**

Yellow powder, yield 63.8 %, ^1^H NMR (600 MHz, DMSO-d_6_): δ 10.92 (s, 1H), 7.83 (dd, J = 13.1, 8.3 Hz, 2H), 7.78 (d, J = 1.6 Hz, 1H), 7.45 (dd, J = 8.6, 2.3 Hz, 1H), 7.39 (t, J = 7.7 Hz, 1H), 7.00 (d, J = 8.1 Hz, 1H), 6.92 (t, J = 7.6 Hz, 1H), 4.22 (s, 2H). ^13^C NMR (151 MHz, DMSO-d_6_): δ 171.38, 163.50, 158.80, 157.96, 135.09, 133.96, 132.16, 131.84, 131.58, 131.00, 129.89, 129.47, 119.91, 117.56, 116.90, 33.86. HRMS [M+H_2_O] (m/z) for C_16_H_11_Cl_2_N_3_O_2_S_2_: calcd. 428.9713, obsd. 428.9940.

**4. N-(2,4-dichlorophenyl)-5-(5-phenyl-4H-1,2,4-thiazolyl)thioacetamide (4)**

Yellow powder, yield 67.6 %, ^1^H NMR (600 MHz, DMSO-d_6_): δ 11.87 (s, 1H), 10.89 (s, 1H), 7.90-7.77 (m, 2H), 7.63-7.53 (m, 2H), 7.36 (t, J = 7.7 Hz, 1H), 6.91 (d, J = 34.7 Hz, 1H), 4.36 (d, J = 15.7 Hz, 1H), 4.25 (d, J = 17.2 Hz, 1H). ^13^C NMR (151 MHz, DMSO-d_6_): δ 170.84, 163.73, 158.21, 157.65, 135.58, 133.59, 133.00, 132.04, 130.27, 129.20, 119.92, 119.86, 117.59, 117.53, 116.70, 29.47. HRMS [M+Na]^+^ (m/z) for C_16_H_11_Cl_2_N_3_O_2_S: calcd. 433.9505, obsd. 433.9440.

**5. N-(4-chloro-2-nitrophenyl)-5-(5-phenyl-4H-1,2,4-thiazolyl)thioacetamide (5)**

White powder, yield 57.5 %, ^1^H NMR (600 MHz, DMSO-d_6_): δ 9.83 (s, 1H), 7.74 (s, 1H), 7.73 (s, 1H), 7.67 (s, 1H), 7.66 (s, 1H), 7.43 (s, 1H), 7.09 (d, J = 8.3 Hz, 2H), 4.08 (s, 2H). ^13^C NMR (151 MHz, DMSO-d_6_): δ 170.58, 157.72, 156.24, 146.02, 136.50, 134.94, 134.10, 133.31, 126.71, 125.79, 125.51, 119.77, 119.43, 117.13, 33.51. HRMS [M+Na]^+^ (m/z) for C_16_H_11_ClN_4_O_4_S_2_: calcd. 444.9808, obsd. 444.0481.

**6. N-(5-chloro-2-nitrophenyl)-5-(5-phenyl-4H-1,2,4-thiazolyl)thioacetamide (6)**

Brown powder, yield 81.4 %, ^1^H NMR (600 MHz, DMSO-d_6_): δ 10.93 (s, 1H), 8.30 (d, J = 8.8 Hz, 1H), 7.91 (d, J = 8.6 Hz, 1H), 7.83 (d, J = 7.7 Hz, 1H), 7.40 (t, J = 7.0 Hz, 1H), 7.02-6.98 (m, 1H), 6.97 (d, J = 8.1 Hz, 1H), 6.92 (t, J = 7.5 Hz, 1H), 4.42 (d, J = 17.5 Hz, 1H), 4.31 (d, J = 17.3 Hz, 1H). ^13^C NMR (151 MHz, DMSO-d_6_): δ 170.50, 163.31, 157.72, 156.19, 144.28, 141.51, 139.66, 139.05, 131.62, 131.00, 129.23, 127.56, 119.44, 117.14, 116.22, 33.52. HRMS [M+Na]^+^ (m/z) for C_16_H_11_ClN_4_O_4_S_2_: calcd. 444.9808, obsd. 444.9692.

**7. N-(2-chloro-4-nitrophenyl)-5-(5-phenyl-4H-1,2,4-thiazolyl)thioacetamide (7)**

Yellow powder, yield 75.8 %,^1^H NMR (600 MHz, DMSO-d_6_): ^1^H NMR (600 MHz, DMSO-d_6_): δ 10.95 (s, 1H), 8.63 (s, 1H), 8.39 (d, J = 8.9 Hz, 1H), 8.03 (d, J = 8.8 Hz, 1H), 7.83 (s, 1H), 7.40 (t, J = 7.2 Hz, 1H), 6.97 (d, J = 8.1 Hz, 1H), 6.92 (t, J = 7.5 Hz, 1H), 4.43 (d, J = 17.1 Hz, 1H), 4.27 (d, J = 17.3 Hz, 1H). ^13^C NMR (151 MHz, DMSO-d_6_): δ 170.44, 157.73, 157.06, 156.28, 146.90, 139.55, 133.58, 131.53, 129.32, 126.58, 126.10, 119.59, 119.44, 117.14, 117.00, 33.55. HRMS [M+Na]^+^ (m/z) for C_16_H_11_ClN_4_O_4_S_2_: calcd. 444.9808, obsd. 444.9676.

**8. N-(4-(trifluoromethoxy)phenyl)-5-(5-phenyl-4H-1,2,4-triazolyl)thioacetamide (8)**

White powder, yield 87.4%, ^1^H NMR (600 MHz, DMSO-d_6_): δ 7.86 (d, J = 8.0 Hz, 1H), 7.59-7.53 (m, 1H), 7.39 (t, J = 7.7 Hz, 1H), 7.00 (d, J = 8.2 Hz, 1H), 6.92 (t, J = 7.5 Hz, 1H), 4.25 (s, 2H). ^13^C NMR (151 MHz, DMSO-d_6_): δ 171.19, 163.31, 157.85, 148.15, 133.83, 133.51, 130.56, 129.32, 121.84, 121.01, 119.34, 117.19, 116.41, 33.32. HRMS [M+Na]^+^ (m/z) for C_17_H_12_F_3_N_3_O_3_S_2_: calcd. 450.0170, obsd. 450.0049.

9. **N-(4-fluorophenyl)-5-(5-phenyl-4H-1,2,4-thiazolyl)thioacetamide (9)**

White powder, yield 56.9%,^1^H NMR (600 MHz, DMSO-d_6_): δ 11.91 (s, 1H), 10.90 (s, 1H), 7.84 (d, J = 7.9 Hz, 1H), 7.45 (dd, J = 8.9, 5.0 Hz, 2H), 7.38 (dt, J = 13.1, 5.5 Hz, 3H), 6.97 (d, J = 8.2 Hz, 1H), 6.93 (t, J = 7.5 Hz, 1H), 4.23 (s, 2H). ^13^C NMR (151 MHz, DMSO-d_6_): δ 171.28, 163.35, 162.66, 161.03, 159.57, 157.78, 133.58, 131.10, 130.64, 129.28, 119.43, 117.16, 116.17, 33.22. HRMS [M+Na]^+^ (m/z) for C_16_H_12_FN_3_O_2_S_2_: calcd. 384.0253, obsd. 384.0151.

10. **N-(3-fluorophenyl)-5-(5-phenyl-4H-1,2,4-thiazolyl)thioacetamide (10)**

White powder, yield 69.7 %, ^1^H NMR (600 MHz, DMSO-d_6_): δ 7.83 (dd, J = 7.9, 1.6 Hz, 1H), 7.58 (dd, J = 15.1, 8.4 Hz, 1H), 7.40-7.36 (m, 1H), 7.36-7.32 (m, 2H), 7.27 (d, J = 8.3 Hz, 1H), 6.98 (d, J = 7.6 Hz, 1H), 6.90 (t, J = 7.2 Hz, 1H), 4.23 (s, 2H). ^13^C NMR (151 MHz, DMSO-d_6_): δ 171.04, 163.30, 162.82, 161.20, 158.07, 136.31, 133.39, 130.75, 129.35, 124.81, 119.11, 117.20, 116.62, 115.94, 115.80, 33.26. HRMS [M+Na]^+^ (m/z) for C_16_H_12_FN_3_O_2_S_2_: calcd. 384.0253, obsd. 384.0161.

11. **N-(2-fluorophenyl)-5-(5-phenyl-4H-1,2,4-thiazolyl)thioacetamide (11)**

White powder, yield 56.9 %, ^1^H NMR (600 MHz, DMSO-d_6_): δ 11.90 (s, 1H), 10.93 (s, 1H), 7.83 (d, J = 8.0 Hz, 1H), 7.57 (dd, J = 12.5, 7.0 Hz, 1H), 7.53 (t, J = 7.5 Hz, 1H), 7.45 (t, J = 9.2 Hz, 1H), 7.39 (d, J = 8.2 Hz, 2H), 6.97 (d, J = 7.9 Hz, 1H), 6.92 (t, J = 7.5 Hz, 1H), 4.39 (d, J = 17.2 Hz, 1H), 4.29 (d, J = 17.2 Hz, 1H). ^13^C NMR (151 MHz, DMSO-d_6_): δ 170.69, 163.43, 158.25, 157.90, 156.54, 133.61, 131.60, 130.94, 129.16, 125.26, 122.23, 119.39, 117.17, 116.62, 116.19, 33.14. HRMS [M+Na]^+^ (m/z) for C_16_H_12_FN_3_O_2_S_2_: calcd. 384.0253, obsd. 384.0145.

12. **N-(2,6-difluorophenyl)-5-(5-phenyl-4H-1,2,4-triazolyl)thioacetamide (12)**

White powder, yield 72.2%, ^1^H NMR (600 MHz, DMSO-d_6_): δ 11.89 (s, 1H), 11.01 (s, 1H), 7.84 (s, 1H), 7.66 (s, 1H), 7.39 (d, J = 9.1 Hz, 3H), 6.97 (s, 1H), 6.92 (t, J = 7.4 Hz, 1H), 4.49 (s, 2H). ^13^C NMR (151 MHz, DMSO-d_6_): δ 170.21, 163.62, 158.96, 158.04, 157.28, 156.93, 133.72, 132.54, 129.16, 119.42, 117.24, 116.12, 112.79, 33.10. HRMS [M+Na]^+^ (m/z) for C_16_H_11_F_2_N_3_O_2_S_2_: calcd. 402.0159, obsd. 402.0032.
